# Addressing Food and Nutrition Security Through Community Initiatives: Assessment of Healthier Food Incentive Programs in U.S. Municipalities

**DOI:** 10.3390/nu18071055

**Published:** 2026-03-26

**Authors:** Nathalie Celestin, Reena Oza-Frank, Brianna Smarsh, Seung Hee Lee, Diane M. Harris

**Affiliations:** 1 Oak Ridge Institute for Science and Education Research Participation Program, Oak Ridge Associated Universities, Oak Ridge, TN 37830, USA; 2 Division of Nutrition, Physical Activity, and Obesity, National Center for Chronic Disease Prevention and Health Promotion, Centers for Disease Control and Prevention, 4770, Buford Highway NE, Mailstop F77, Atlanta, GA 30341, USAhva6@cdc.gov (D.M.H.); 3 Division of Overdose Prevention, National Center for Injury Prevention and Control, Centers for Disease Control and Prevention, Atlanta, GA 30341, USA

**Keywords:** vegetables, fruit, nutrition, community nutrition, food assistance, diet, food security, nutrition policy

## Abstract

**Background/Objectives:** Healthy food incentive programs (HFIP), such as fruit and vegetable voucher incentives, can supplement other nutrition assistance programs to support food and nutrition security. However, little is known about the prevalence of HFIP, particularly at the municipal level. This study examines the prevalence of HFIP in a nationally representative sample of U.S. municipalities and the association between the availability of HFIP and municipal characteristics. **Methods:** Using data from the CDC’s 2021 National Survey of Community-Based Policy and Environmental Supports for Healthy and Active Living (*n* = 1982 municipalities), a weighted bivariate analysis and multivariable logistic regression analysis were conducted to estimate the prevalence of HFIP overall and by municipal characteristics, and to assess the relationship between municipal characteristics and HFIP. **Results:** Only 7.8% of municipalities reported offering HFIP in 2021. The odds of having an HFIP were higher in municipalities with a food policy council (aOR 2.8; 95%CI: 1.9, 3.9) compared to those without. Larger communities (size ≥ 50,000 reported 24.6%) and those with a higher prevalence of residents living in poverty were also more likely to offer HFIP. **Conclusions:** Few municipalities reported offering HFIP. Results suggested that engaging institutions and individuals (e.g., via food access coalitions) may be strategies that could support municipalities initiating and implementing HFIP to improve diet quality and reduce chronic disease risk.

## 1. Introduction

Lack of affordability and accessibility to healthy foods remains a barrier globally and for many Americans. Healthy food incentive programs (HFIP), such as fruit and vegetable voucher incentives, can expand nutrition assistance benefits to support diet quality and nutrition security [1] by mitigating the increased costs associated with purchasing healthy foods [2]. Food security is defined as access to enough food for an active, healthy life at all times [3], while nutrition security is the consistent and equitable access to healthy, safe, affordable foods essential for optimal health and well-being [4]. Most evidence of global HFIP shows these programs increase the purchase and consumption of healthier, subsidized foods, improve food security, and enhance nutrition knowledge, despite mixed results on diet quality indicators, physical health outcomes, and mental health [5].

In the U.S. in 2023, 13.5% of households were food insecure [6]. No national surveillance currently exists for the construct of nutrition security. To address food and nutrition security, the Supplemental Nutrition Assistance Program (SNAP) supplements a household’s income to allot specific funds towards the purchase of food at authorized retailers (such as convenience stores, farmers markets, grocery stores, and supermarkets) across the country using electronic benefits transfer (EBT) systems [7]. In 2023, approximately 51.9% of households experiencing food insecurity received SNAP benefits [6]. While SNAP benefits relieve some burden for household food spending, HFIPs are voluntary state and local level programs independent of SNAP that can supplement SNAP benefits to encourage the purchase of healthier foods (e.g., fruits, vegetables) among eligible participants [8].

Research has shown that HFIPs are an effective way to increase access, affordability, and consumption of healthier foods to improve diet quality as well as support household economic security [9,10]. HFIP provides vouchers, coupons, or cash incentives that are redeemable at various retail sites such as convenience stores, farmers’ markets, and grocery stores [8]. Funding to support HFIP can come from multiple sources, including federal funding, state and local municipal funding, and/or philanthropy. However, implementation of HFIP often varies by locality and program type [10]. Differences in program implementation may include types of retail outlets, geographic focus, redemption methods, incentive matching amount, and foods eligible for redemption [10]. Although these differences are important, little is known about the prevalence of HFIP and differences in HFIP at the municipal level, which could provide insights into HFIP uptake among communities. This study aimed to estimate the prevalence of HFIP in municipalities with ≥1000 persons and assess the association between municipality characteristics and the availability of HFIP for SNAP recipients using a national survey conducted in 2021. HFIP in this study refers to incentive programs that allow SNAP recipients to purchase healthier foods at a subsidized or discounted rate beyond standard SNAP benefits.

## 2. Materials and Methods

### 2.1. Survey and Study Sample

The 2021 National Survey of Community-Based Policy and Environmental Supports for Healthy Eating and Active Living (CBS HEAL) survey is a modified 7-section, self-reported questionnaire modeled after a pilot (2012) and previous cycle (2014) [11,12]. Conducted from May to September 2021, the survey gathered information about existing municipal-level programs and policies that support healthy eating and active living. Methods for the 2021 survey have been previously published [10]. Surveys were distributed to a stratified sample (*n* = 4417) representative of the United States. Municipalities with fewer than 1000 persons were excluded from the study, as a 2012 pilot found these municipalities were less likely to have surveyed existing policies [11]. Stratification criteria included geographic region, percent urbanicity, and population size. A letter was mailed to a primary respondent, the city or town manager, city planner, city administrator, or a person with similar responsibilities within each municipality. The letter detailed the scope of the study and provided the respondent with a unique identification number and access to the study webpage. Reminders were delivered electronically and by mail from weeks 3 to 17. During the follow-up period, nonrespondents were mailed a paper survey with the option to return using the postage-paid business reply envelope or email a scanned copy to the project staff for data entry. In 2021, 1982 municipalities completed the survey, corresponding to a response rate of 45%. The Centers for Disease Control and Prevention (CDC) and National Opinion Research Center (NORC) at the University of Chicago both determined that the survey was not human subjects research and did not need IRB review.

### 2.2. Measures

Presence of HFIP was assessed by the survey question, “Does your local government offer incentive programs that allow Supplemental Nutrition Assistance Program (SNAP) recipients to purchase healthier foods at a subsidized or discounted rate beyond standard SNAP benefits?” Response options were “yes”, “no”, “don’t know”, and for the present analysis, responses were dichotomized into having HFIP and not having HFIP (combined “no” and “don’t know”).

Explanatory variables were municipality-level characteristics: population size (<2500; 2500–49,999; ≥50,000), urbanicity (urban; rural), census region (Northeast, Midwest, South, West), poverty prevalence (<20% below FPL; ≥20% below FPL), race and ethnicity (≤50% Non-Hispanic White; >50%% Non-Hispanic White) and presence of a food policy council or coalition (yes; no). Municipal population size was obtained from the 2017 Census of Governments [13]. Urbanicity was classified using the 2010 U.S. Census Urban Area to Place Relationship File; municipalities were categorized as urban if >50% of the population resided in areas defined as urban [14]. The 2020 American Community Survey 5-Year Estimates were used to define poverty prevalence and racial and ethnic composition [15]. The presence of a food policy council (FPC) was determined based on responses received for the CBS HEAL survey question that asked, “Does your community have a local, county, or regional food policy council, food security coalition, or other community group working to increase access to healthy food?” Response options were “yes”, “no”, and “don’t know”. For this analysis, responses were dichotomized into having a council and not having a council (combined “no” and “don’t know”). The percentage of “don’t know” responses was 18.2%. To investigate the potential impact of differing patterns of “don’t know” responses on results, we also performed sensitivity analyses where “don’t know” responses to the HFIP question were excluded from calculations of overall prevalences and related statistical tests.

### 2.3. Statistical Analysis

Descriptive statistics were calculated to describe the municipality characteristics of the sample. A weighted bivariate analysis was conducted to assess the prevalence (%) of HFIP by municipality characteristics: population size, urbanicity, census region, poverty prevalence, racial/ethnic composition, and presence of FPC. Chi-squared tests were conducted to assess statistical differences in the presence of HFIP by municipality characteristics.

A weighted multivariable logistic regression analysis was conducted to examine independent associations between the availability of HFIP and municipality characteristics, with *p* < 0.05 considered statistically significant. Data was analyzed using SAS version 9.4 (SAS 9.4, Cary, NC, USA. SAS Institute Inc.). Analyses accounted for the survey design and nonresponse.

## 3. Results

Of the 1982 municipalities that responded, 58.6% had a population size of 2500–49,999, 75.5% were urban, and 36.2% were in the South region. Approximately 80% had a poverty prevalence of <20%, and 82.3% had a racial and ethnic distribution of >50% non-Hispanic White. Among municipality respondents, 72.4% reported that they did not have an FPC present to increase healthy food access (Table 1).

Overall, 7.8% of municipalities reported having HFIP available in 2021 (Table 2). The prevalence of having HFIP varied by municipality characteristics. HFIP were more common in municipalities with population size ≥ 50,000 (24.6% vs. 7.0% in 2500–49,999 and 5.4% in 1000–<2500 population size [*p* < 0.05]), ≥20% of population below FPL (12.2% vs. 6.6% in municipalities with <20% below FPL [*p* < 0.05], ≤50% of residents being Non-Hispanic White (13.2% vs. 6.7% in municipalities with >50% Non-Hispanic White [*p* < 0.05]) and FPC working to increase healthy food access (14.9% vs. 5.0% in municipalities without a FPC [*p* < 0.05]).

Based on the multivariable analysis, municipalities with a population size of 2500–49,999 (aOR 0.3; 95% CI: 0.2, 0.5) or 1000–<2500 (aOR 0.2; 95% CI: 0.1, 0.4) had lower odds of having HFIP compared to municipalities with a population size of ≥50,000. In contrast, the odds of having HFIP were nearly double in municipalities with ≥20% of residents below FPL (aOR 1.9; 95% CI: 1.2, 2.8) vs. <20% of residents below FPL and nearly triple in municipalities with an FPC (aOR 2.8; 95%CI: 1.9, 3.9) compared to municipalities without an FPC. In sensitivity analyses where “don’t know” responses to the HFIP question were excluded from overall prevalence estimates, χ^2^ tests, and logistic regressions, results remained similar.

## 4. Discussion

In 2021, only 7.8% of municipalities reported having HFIP. After adjustment for covariates, the odds of having an HFIP was higher among municipalities with ≥20% of population below FPL, municipalities with a population size ≥ 50,000, and municipalities with an FPC. Areas where there is a high proportion of residents with low incomes typically also have a high percentage of people who may be eligible for SNAP, and thus eligible for HFIP as well [16]. For context, a 2011 study found SNAP stakeholders identified the high cost of healthy foods as one of the top three barriers to improving nutrition for SNAP participants [17]. HFIP increases the purchasing power of SNAP participants by reducing cost barriers associated with purchasing healthy foods [10]. In a randomized controlled trial, incentive benefits significantly increased fruit and vegetable intake among SNAP households who received a 30% rebate (*n* = 7500) compared to those who were allotted their standard SNAP benefits (*n* = 47,595) [9]. The diets of these participants also had higher Healthy Eating Index-2010 scores [9]. GusNIP nutrition incentive participants reported improvements in their diet and perceived health [16]. Individuals who participated in a nutrition incentive program (*n* = 7070) for six months or more from August 2021 to September 2022, the same time period of this study, reported “very good” and “excellent” health at a higher rate (27.2%) than first-time participants (21.5%) [16].

In this study, the availability of HFIP was much more common in municipalities with an FPC. This finding was not surprising as FPCs are networks of cross-sector collaborators who work together to address local, regional, or state-level food system needs [18]. Like our findings, previous studies showed that the presence of FPCs was significantly associated with municipal-level policies or practices to improve access to healthy foods [19].

Based on a narrative review of global studies on HFIPs, healthy food benefits may effectively increase the purchase and consumption of subsidized items, resulting in positive outcomes in various countries [5]. Additionally, studies show improvements in food security indicators and enhanced nutrition knowledge among participants [5]. However, barriers such as limited enrollment options, methods of provision for electronic benefits, benefit amounts, and diverse eligible healthy foods may affect the ability of these programs to achieve positive nutrition-related outcomes [5]. When designing and implementing HFIPs, community leaders and policymakers should account for these barriers to address the unique needs and preferences of their communities.

### Limitations

Our findings are subject to some limitations. First, municipalities with <1000 people were excluded from this study, which may limit generalizability to small municipalities. Although the survey pilot revealed that municipalities with <1000 people were least likely to have such policies and only accounted for 3% of the US municipal population [11], targeted research into these smaller, often rural, communities, which may face different logistical barriers to implementing HFIP compared to the larger municipalities, could provide useful information on program implementation. Second, study responses were self-reported by a municipal official or similar staff. Responses were not verified with written policies or practices, and there may be variation in the respondents’ knowledge and understanding of municipal planning and/or policies related to HFIP to increase food access. Future studies might explore how to verify these responses against written policies or legal databases to mitigate the risk that respondents may have been unaware of existing local policies. Third, “don’t know” responses were coded as “no”. Therefore, it is possible that prevalence estimates may underestimate the actual prevalence of availability of incentive programs; some respondents who replied “don’t know” may in fact have HFIP available in their municipality or have other types of incentive programs. Based on the respondents’ interpretation of the question, there may be variations in their responses about the inclusion of programs such as produce prescription programs, which also include an incentive component that may or may not be linked to SNAP participation. If respondents included programs such as produce prescription programs in their responses, the estimates of this study would be higher than if those programs were not included. The survey did not collect data on HFIP design (e.g., incentive amounts, eligible retailers, funding sources), limiting insights into factors associated with program adoption, which would be helpful information for community leaders and policymakers in designing community HFIP. The survey also did not collect data on potential confounders that may have impacted results, such as municipal budgets or state-level HFIP mandates. Finally, this study is cross-sectional and therefore no causal relationships can be determined.

## 5. Conclusions

HFIP has the potential to increase FV intake and reduce food and nutrition insecurity by increasing household purchasing power for fruits and vegetables. These results can serve as a baseline for monitoring progress towards increasing the availability of healthy incentive programs offered by municipalities in the U.S. Municipalities seeking to establish or expand the availability of HFIP may consider strengthening community-based partnerships to increase awareness and access to healthy food. Furthermore, public health practitioners can support implementation of HFIPs by conducting landscape assessments of existing programs, convening cross-sectoral partners to build capacity, securing complementary funding and leveraging policy opportunities, improving and streamlining processes and technology (e.g., EBT or retail technology), and conducting evaluation and disseminating findings.

## Figures and Tables

**Table 1:** Municipality Characteristics, National Survey of Community-Based Policy and Environmental Supports for Healthy Eating and Active Living (CBS-HEALS), 2021.

Municipality Characteristics	*N*	Weighted %
Overall	1982	100
Population		
1000–<2500	675	33.9
2500–49,999	1141	58.6
≥50,000	166	7.5
Rural/Urban Status (*n* = 1974)		
Rural	519	24.5
Urban	1455	75.5
Census Region		
Northeast	304	14.2
Midwest	661	35.0
South	566	36.2
West	451	14.6
Poverty Prevalence		
<20% below FPL	1582	78.7
≥20% below FPL	400	21.3
Race and Ethnicity		
>50% Non-Hispanic White	1656	83.2
≤50% Non-Hispanic White	326	16.8
Presence of food policy council working to increase healthy food access ^a^ (*n* = 1968)		
Yes	548	27.6
No	1420	72.4

^a^ The presence of a food policy council was determined based on responses received for the CBS HEAL survey question that asked, “Does your community have a local, county, or regional food policy council, food security coalition, or other community group working to increase access to healthy food?” Response options were “yes”, “no”, and “don’t know”. For this analysis, responses were dichotomized into having a council and not having a council (combined “no” and “don’t know”).

**Table 2:** Prevalence (%) of Healthy Food Incentive Programs by Municipality Characteristics (Bivariate Analysis) and Association Between Municipality Characteristics and Availability of Incentive Programs for SNAP Recipients (Multivariate Analysis), United States, 2021.

Municipality Characteristics	Prevalence of HFIP ^a^		Multivariable Analysis (*n* = 1940)
	Yes	No & Don’t Know	aOR (95% CI)
	Weighted %	Weighted %	
Overall (*n* = 1952)	7.8	92.2	
Population (*n* = 1952) ^c^			
1000–<2500	5.4	94.6	0.2 (0.1, 0.4)
2500–49,999	7.0	93.0	0.3 (0.2, 0.5)
≥50,000	24.6	75.4	Reference
Rural/Urban Status (*n* = 1944)			
Urban	8.1	91.9	Reference
Rural	6.5	93.5	1.5 (0.8, 2.9)
Census Region (*n* = 1952)			
Northeast	7.4	92.6	0.9 (0.5, 1.6)
Midwest	7.2	92.8	Reference
South	8.3	91.7	0.8 (0.5, 1.3)
West	8.3	91.7	0.8 (0.5, 1.3)
Poverty Prevalence (*n* = 1952) ^c^			
<20% below FPL	6.6	93.4	Reference
≥20% below FPL	12.2	87.8	1.9 (1.2, 2.8)
Race and Ethnicity (*n* = 1952) ^c^			
>50% Non-Hispanic White	6.7	93.3	Reference
≤50% Non-Hispanic White	13.2	86.8	1.3 (0.8, 2.1)
Presence of food policy council working to increase healthy food access ^b^ (*n* = 1948) ^c^			
Yes	14.9	85.1	2.8 (1.9, 3.9)
No	5.0	95.0	Reference

^a^ Availability of HFIP was assessed by the survey question, “Does your local government offer incentive programs that allow Supplemental Nutrition Assistance Program (SNAP) recipients to purchase healthier foods at a subsidized or discounted rate beyond standard SNAP benefits?” Response options were “yes”, “no”, “don’t know”. For the present analysis, responses were dichotomized into having HFIP and not having HFIP (combined “no” and “don’t know”). ^b^ Presence of a food policy council was determined based on responses for the CBS HEAL survey question that asked, “Does your community have a local, county, or regional food policy council, food security coalition, or other community group working to increase access to healthy food?” Response options were “yes”, “no”, “don’t know”. For this analysis, responses were dichotomized into having a council and not having a council (combined “no” and “don’t know”). ^c^ Statistical significance (*p*-value < 0.05) based on a Chi-square test.

## Data Availability

Data available at https://data.cdc.gov/Nutrition-Physical-Activity-and-Obesity/National-Community-Based-Survey-of-Supports-for-He/8hus-y5nc/about_data (accessed on 23 March 2026).
